# A case of solitary bone plasmacytoma to multiple myeloma: a case report

**DOI:** 10.3389/fmed.2026.1774242

**Published:** 2026-02-25

**Authors:** Zhimin Fang, Zhong Tian, Jiyang Tang, Bo Lei, Zhongcong He, Cheng Zhu, Huangyu Luo, Ni Fu, Yuguang Shen

**Affiliations:** 1 Department of Thoracic Surgery, The Third Affiliated Hospital of Zunyi Medical University, Zunyi, China; 2 Department of Urology, The Second Affiliated Hospital of Zunyi Medical University, Zunyi, China

**Keywords:** mm, rib, SBP, SEP, SP

## Abstract

Solitary bone plasmacytoma of the rib is uncommon but recognized. We report a case presenting with recurrent, predominantly nocturnal chest wall pain. Imaging revealed an expansile lesion on the left ninth rib and initially suggested fibrous/osteofibrous dysplasia. The patient underwent resection of the involved rib segment. Histopathology and immunohistochemistry confirmed solitary bone plasmacytoma. The patient declined the recommended adjuvant radiotherapy. Twenty-one months later, the disease progressed to multiple myeloma.

## Introduction

Solitary plasmacytoma (SP) is a rare plasma cell neoplasm that presents as a single lesion and is classified as either solitary bone plasmacytoma (SBP) or solitary extramedullary plasmacytoma (SEP) (1, 2). Rib involvement in SBP is uncommon. We report a patient who presented with recurrent chest wall pain of unclear origin. Imaging demonstrated an expansile lesion in the left ninth rib, and the patient underwent en bloc resection of the involved rib segment. Histopathology with immunohistochemistry confirmed SBP of the rib. The patient declined adjuvant radiotherapy, and no systemic therapy was administered. Twenty-one months postoperatively, the patient developed left-sided low back pain and was diagnosed with multiple myeloma (MM). This case underscores the risk of progression from SBP to MM.

## Case report

A 64-year-old woman presented with a 1-year history of intermittent left chest wall pain that she had self-managed with analgesics. The current episode lasted 1 week without precipitating trauma, palpitations, or dyspnea. On examination, she had focal tenderness over the left ninth rib; cardiopulmonary findings were unremarkable, and routine laboratory tests were within normal limits (Table 1).

**Table 1:** Laboratory studies.

Laboratory study	Result	Laboratory study	Result
Complete blood test		Anti-HIV antibody	Negative
Hemoglobin, g/L	121	Syphilis antibody	Negative
Total leukocyte count, × 10^9^/L	6.6	HBs antigen	Negative
Neutrophil count	4.2	Anti-HCV antibody	Negative
Lymphocyte count	1.9	Immunoglobulin	
Platelet count, × 10^3^/L	229	IgG, g/L	11.40
TB-IgG	Negative	IgA, g/L	1.80
ESR, mm/h	7.8	IgM, g/L	0.74
Blood urea nitrogen, mg/dL	31.25	Ig light chain-kappa, g/L	
Serum creatinine, mg/dL	0.56	Ig light chain-lambda, g/L	8.30
AST, U/L	21	CA125 II, U/mL	
ALT, U/L	22	CA19-9, U/mL	5.43
Blood albumin, g/L	24.1	AFP, ng/ml	13.7
Globulin, g/L	32.6	CEA, ng/ml	5.6
Prealbumin	203.6	Syphilis antibody	6.03
A/G	1.5	HBs antigen	1.6

**Figure 1:**
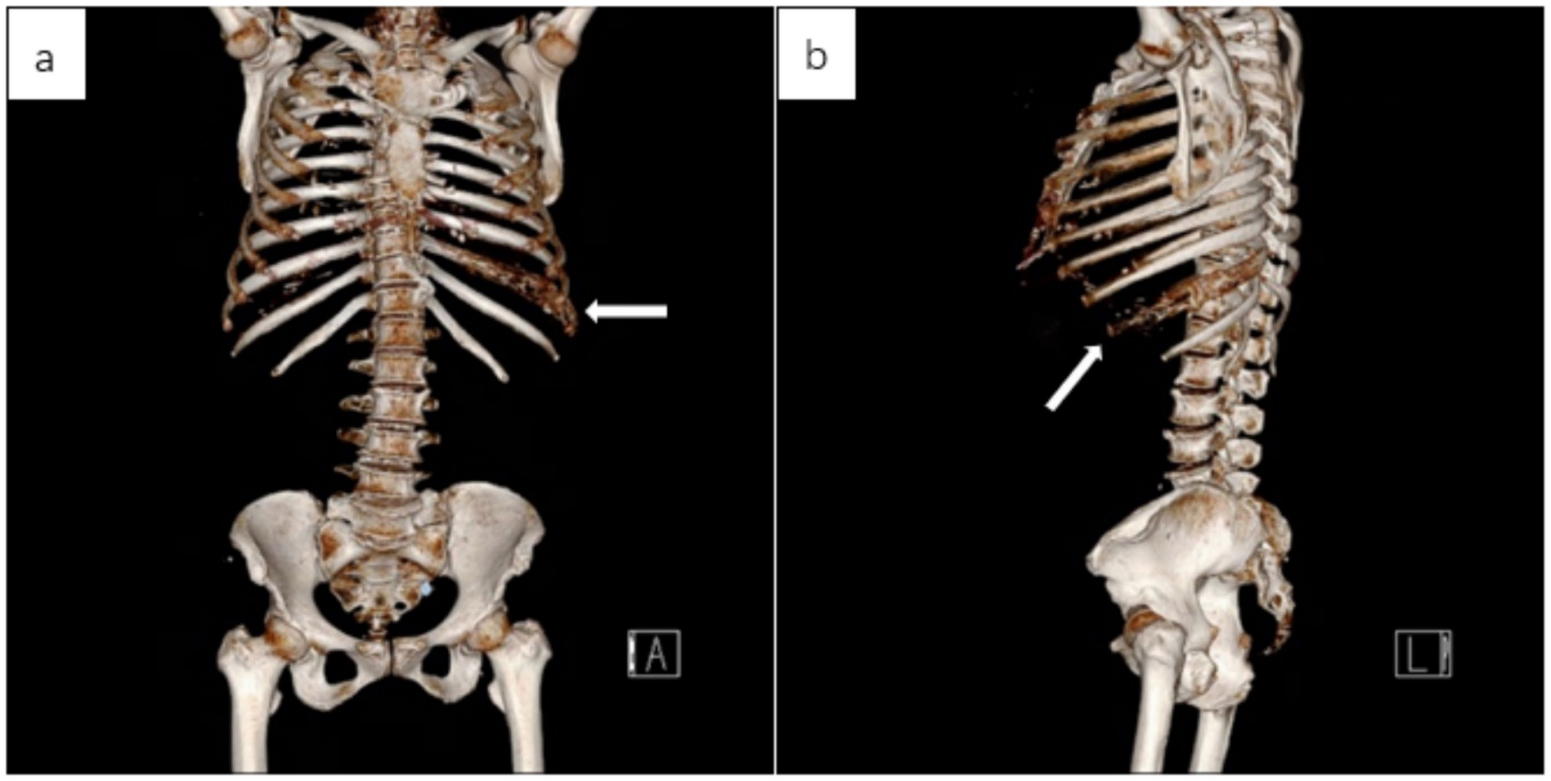
CT (bone window) of the chest, abdomen, and pelvis demonstrates an expansile osteolytic lesion of the left ninth rib: **(a)** Coronal reformatted image and **(b)** sagittal reformatted image.

**Figure 2:**
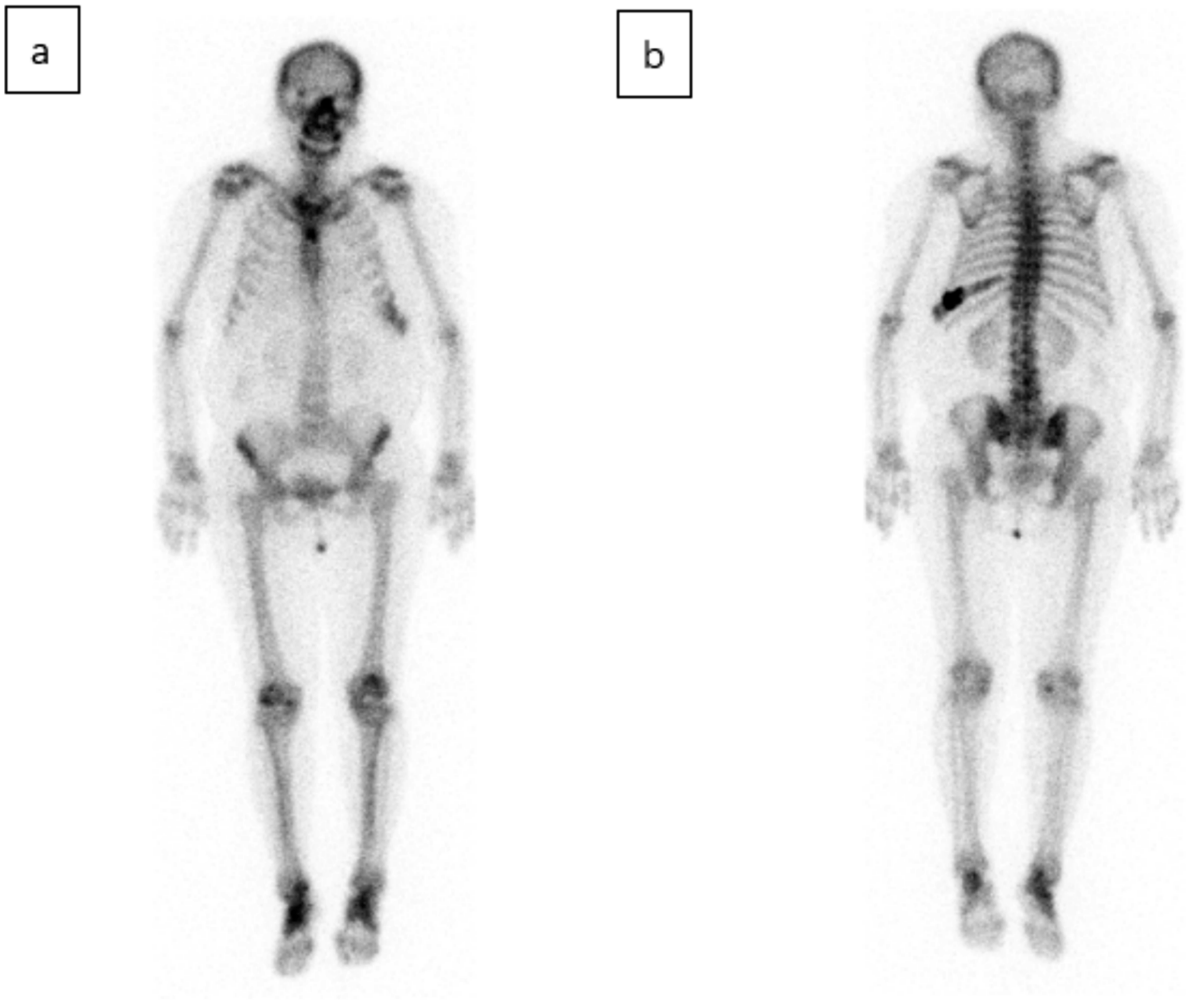
Whole-body ^99m^Tc-MDP bone scintigraphy demonstrates focal, intense radiotracer uptake in the left ninth rib, corresponding to the expansile rib lesion seen on CT.

CT of the chest, abdomen, and pelvis (bone windows) showed an expansile lesion in the left ninth rib (Figure 1). Osteofibrous dysplasia was initially considered, and a bone scan demonstrated focal increased uptake in the same rib (Figure 2). After admission and exclusion of surgical contraindications, she underwent en bloc resection of the left ninth rib lesion.

**Figure 3:**
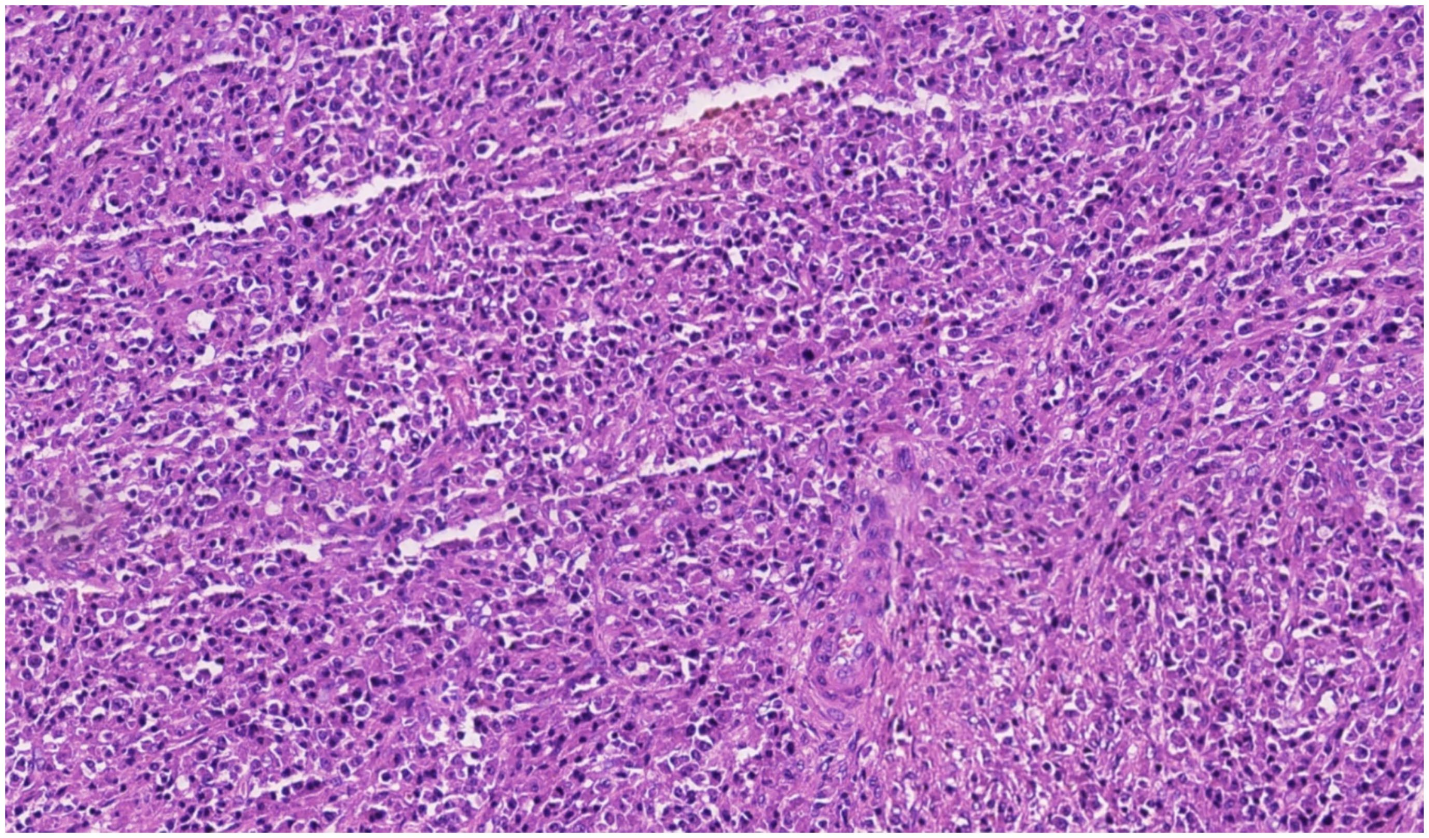
Histopathology of the rib lesion shows dense sheets of plasma cells with eccentric nuclei, clumped chromatin, and a perinuclear hof, consistent with a monoclonal plasma cell infiltrate (H&E, original magnification ×20).

**Figure 4:**
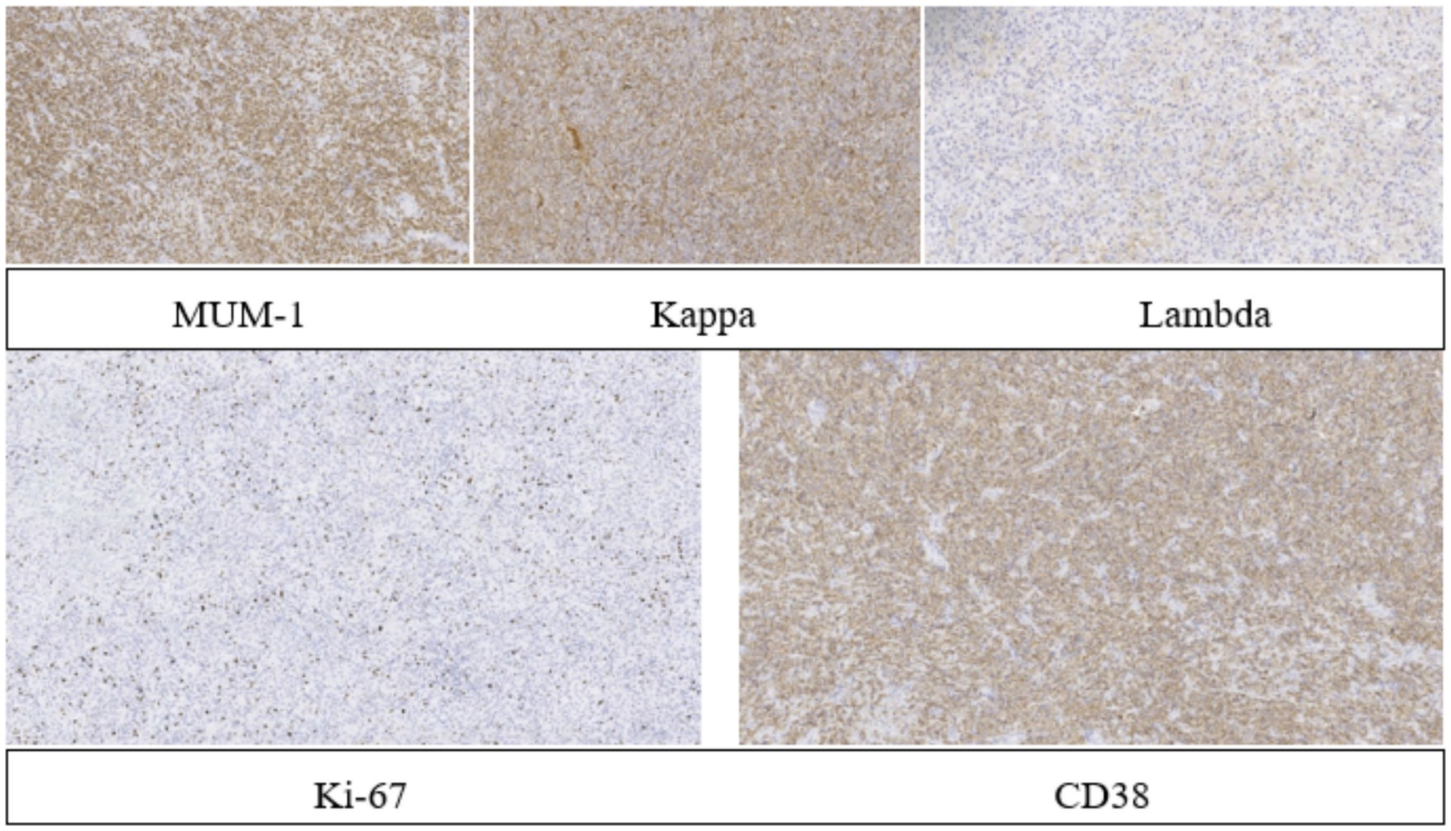
Immunohistochemistry of the rib lesion demonstrates plasma cells positive for MUM1 and CD38, with cytoplasmic kappa and lambda light-chain expression; Ki-67 proliferation index is approximately 10%.

Histopathology (Figure 3), together with immunohistochemistry, supported a plasma cell neoplasm: Tumor cells were positive for MUM1, Kappa, Lambda, and CD38 with a Ki-67 index of approximately 10% (Figure 4); they were negative for CD19, CD20, CD3, CD5, CD34, CD68, CD56, SMA, EMA/S-100, chromogranin A, desmin, MyoD1, and melan-A. Surgical margins and sampled lymph nodes were free of tumor. Bone marrow cytology showed active hematopoiesis. Integrating the morphologic and immunophenotypic findings, a diagnosis of SBP (left ninth rib) was established. The patient declined adjuvant radiotherapy and received no systemic therapy.

**Figure 5:**
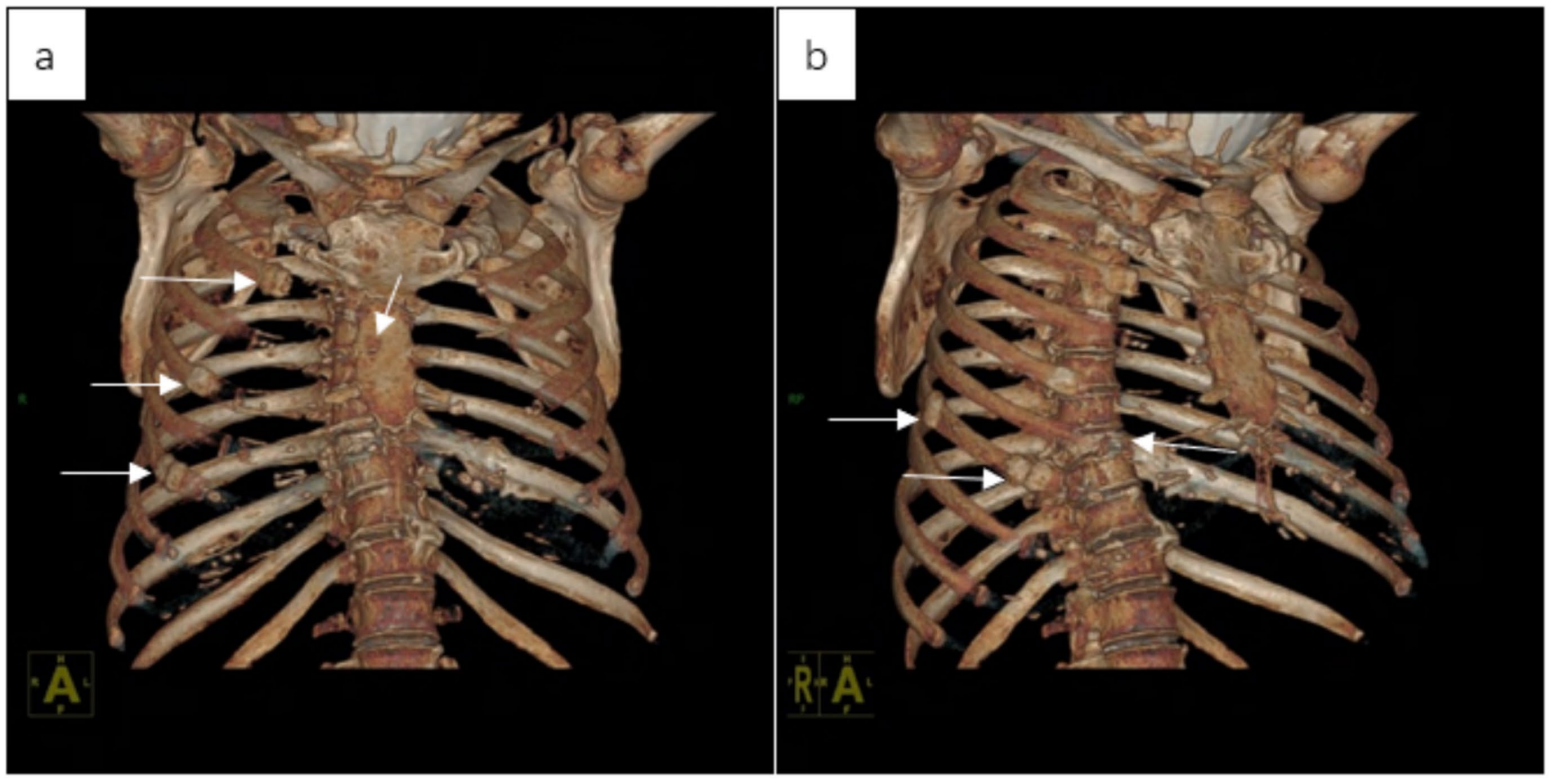
CT (bone window) of the chest and thoracic spine demonstrates postoperative absence of the left ninth rib consistent with prior resection. **(a)** Coronal image shows multifocal osteolytic lesions of the sternum and multiple bilateral ribs with pathologic fractures and callus formation. **(b)** Sagittal image shows vertebral osteolysis with compression fractures.

**Figure 6:**
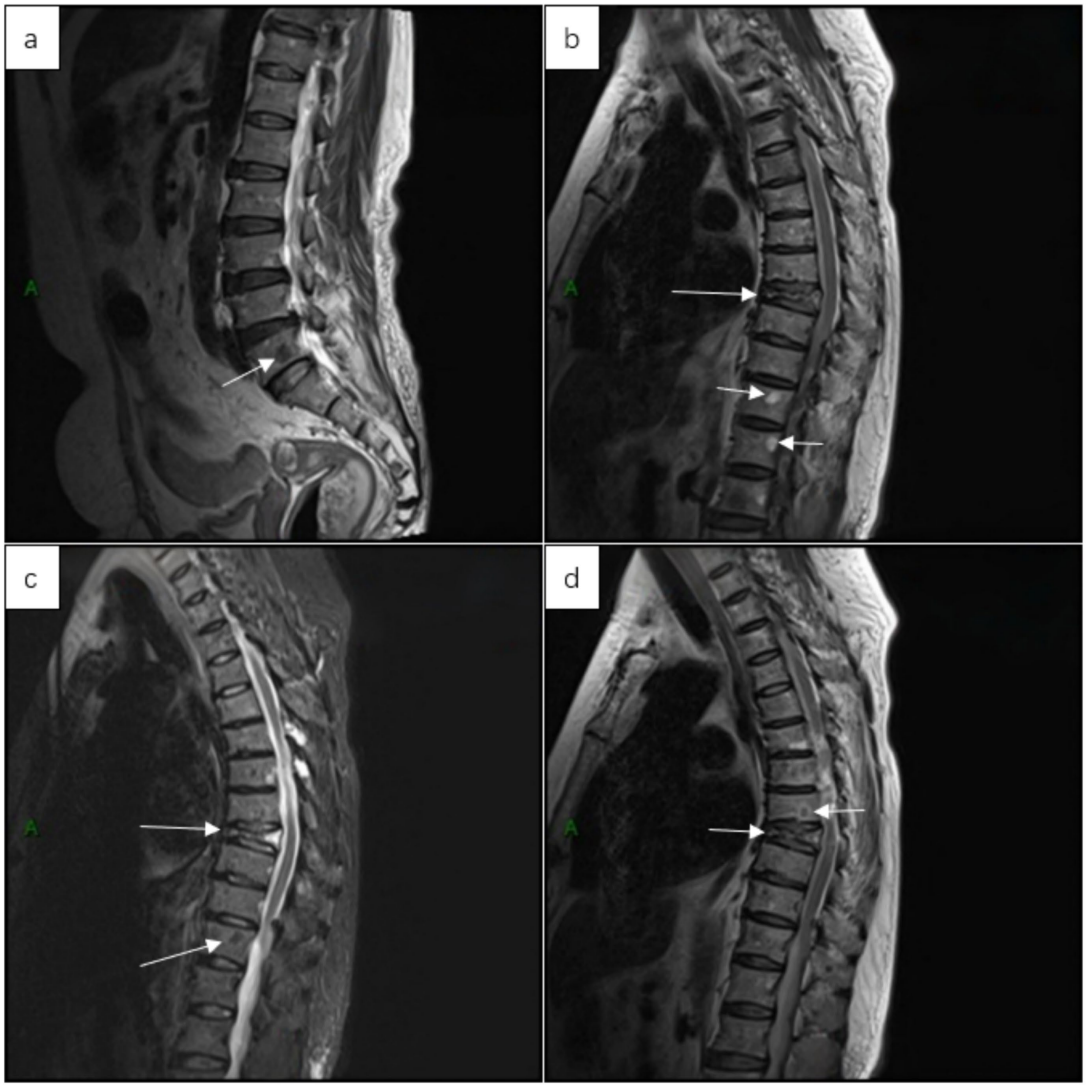
MRI of the spine demonstrating multifocal osseous lesions involving the lumbar spine **(a)** and thoracic spine **(b–d)**, consistent with myelomatous infiltration, with partial compression deformities in several thoracic vertebral bodies.

At 21 months postoperatively, she developed left-sided low back pain. CT demonstrated osteolytic lesions involving the sternum, multiple thoracic vertebrae, and bilateral ribs (Figure 5), and spine MRI confirmed multifocal vertebral marrow replacement consistent with MM (Figure 6). CT-guided vertebral biopsy showed a plasma cell neoplasm with positivity for MUM1, Kappa, Lambda, and CD38 with a Ki-67 index of approximately 10%, confirming MM. She subsequently started lenalidomide-based therapy. At the time of manuscript revision (26 months after surgery), the patient is alive.

## Discussion

Solitary plasmacytomas (SPs) are rare plasma cell neoplasms with an annual incidence of approximately 0.15 per 100,000, accounting for approximately 5% of plasma cell disorders and showing a male predominance of approximately 2:1 (1). By anatomic site, SPs are classified as SBP or SEP (3). SBP constitutes approximately 60–70% of cases and typically presents with localized bone pain, often worse at night; pathologic fractures; and, for spinal lesions, neurologic compromise due to cord or nerve root compression may occur. SEPs most commonly arise in the upper aerodigestive tract and present as localized masses with or without pain; site-related symptoms may include nasal obstruction or discharge, epistaxis, sinus pressure, dysphagia, hoarseness, dyspnea, or a globus sensation (4).

SBP arises preferentially in red marrow-rich bones, most commonly within the axial skeleton—vertebrae, pelvis, ribs, and skull. Mandibular/maxillary involvement is uncommon (approximately 4%) (5). Rib lesions are rare but well-documented, with only sporadic case reports and small series published. Our case adds to this limited literature.

SBP and MM typically present with purely osteolytic bone destruction, so MDP bone scintigraphy cannot serve as a standalone diagnostic or exclusion criterion (6). In this study, MDP imaging showed abnormal rib lesions but neither confirmed SBP nor provided a basis for staging. Tracer uptake within lesions may reflect reactive bone remodeling and associated osteoblastic activity. Tc-99 m MDP enables whole-skeleton imaging in a single session, allowing rapid detection and delineation of disease extent and identification of targets for subsequent evaluations and treatments. Tc-99 m MDP can also be used for post-therapy follow-up (7).

The diagnosis of SP requires histologic confirmation of a clonal plasma cell infiltrate (light-chain restriction by IHC/ISH or flow cytometry), bone marrow evaluation to exclude MM (no clonal plasma cells, or <10% for SP with minimal marrow involvement), absence of myeloma-defining events (CRAB or SLiM criteria), and whole-body imaging—preferably FDG-PET/CT or whole-body MRI (or low-dose whole-body CT)—to exclude additional lesions (8). Definitive radiotherapy is the first-line treatment and achieves high local control; typical doses are 40–50 Gy to the involved site with appropriate margins (9, 10). Surgery is reserved for tissue diagnosis, mechanical stabilization, or urgent decompression; when undertaken for SBP, adjuvant radiotherapy is generally recommended to optimize local control. Regular post-treatment surveillance with serum/urine monoclonal protein studies, serum free light chains, and symptom-directed imaging is advised.

Most patients with SP achieve durable local control after guideline-concordant therapy, yet progression to MM remains at substantial risk (11, 12). Long-term series indicate that approximately 50% of SBP and approximately 25–35% of SEP progress to MM within 10 years of diagnosis (13–15). Higher risk is associated with minimal marrow involvement detected by sensitive flow cytometry, persistence or rise of serum/urine M-protein after radiotherapy, an abnormal serum free light-chain ratio, tumor size >5 cm, multiple foci on MRI/PET, and older age. Our patient progressed to MM 21 months after diagnosis, underscoring the importance of guideline-concordant local therapy and vigilant, standardized follow-up with serial monoclonal protein studies, serum free light chains, and symptom-directed imaging (1).

MM is a clonal plasma cell malignancy characterized by bone marrow infiltration of abnormal plasma cells and overproduction of monoclonal immunoglobulin (M-protein) or free light chains (16). Clinically, MM is associated with myeloma-defining organ damage—CRAB features: hypercalcemia, renal insufficiency, anemia, and osteolytic bone disease (13, 17). Per IMWG criteria, MM is diagnosed by ≥10% clonal bone marrow plasma cells or a biopsy-proven plasmacytoma plus at least one myeloma-defining event (CRAB or SLiM biomarkers: ≥60% clonal plasma cells, involved/uninvolved free light-chain ratio ≥100 with involved FLC ≥ 100 mg/L, or >1 focal lesion on MRI); imaging commonly demonstrates multiple osteolytic lesions (18).

Contemporary management of MM is risk-adapted and built on combination regimens incorporating a proteasome inhibitor, an immunomodulatory drug, and dexamethasone, with early autologous stem-cell transplantation (ASCT) for eligible patients (19). Common frontline regimens include bortezomib-lenalidomide-dexamethasone (VRd) and daratumumab-containing quadruplets (e.g., Dara-VRd). For transplant-ineligible patients, effective options include daratumumab-lenalidomide-dexamethasone (D-Rd) or bortezomib-melphalan-prednisone (VMP) and its daratumumab-based variant (D-VMP). Historically, the MP regimen refers to melphalan plus prednisone and has largely been supplanted by triplet/quadruplet therapy.

Targeted agents include the proteasome inhibitors bortezomib, carfilzomib, and ixazomib, and the immunomodulatory drugs lenalidomide, thalidomide, and pomalidomide. Immunotherapies for relapsed/refractory disease encompass anti-CD38 monoclonal antibodies (daratumumab, isatuximab), BCMA-directed CAR-T cells (idecabtagene vicleucel, ciltacabtagene autoleucel), bispecific antibodies (e.g., teclistamab, elranatamab, GPRC5D-targeted talquetamab), and the anti-BCMA antibody–drug conjugate belantamab mafodotin (20, 21).

Lenalidomide maintenance after initial therapy prolongs progression-free and overall survival, and pomalidomide-based combinations improve outcomes in relapsed disease. Incorporation of anti-CD38 antibodies increases the depth of response and rates of minimal residual disease negativity.

Future directions in MM emphasize rational, biomarker-guided combination therapies to deepen responses and delay resistance, including next-generation immunomodulators, proteasome inhibitors, and T-cell–redirecting agents (anti-BCMA or anti-GPRC5D CAR-T cells and bispecific antibodies), with dual-target strategies to reduce antigen escape. Personalization will leverage cytogenetic/molecular profiling [e.g., del(17p), t(4;14), 1q gain, and t(11;14) guiding venetoclax use] and dynamic biomarkers such as minimal residual disease to enable response-adapted escalation or de-escalation of therapy (19, 22).

We report a rare case of SBP of the rib that progressed to MM 21 months after diagnosis. This case highlights the need to consider plasma cell neoplasms in patients with destructive rib lesions and to pursue a comprehensive workup—serum and urine protein electrophoresis with immunofixation, serum free light chains, tissue biopsy with confirmation of clonality, bone marrow evaluation, and whole-body imaging (FDG-PET/CT or whole-body MRI)—to avoid misdiagnosis, secure a definitive diagnosis, and deliver guideline-concordant therapy and follow-up aimed at optimizing local control and delaying progression.

## Conclusion

Progression from SP to MM is a recognized and clinically important risk, underscoring the need for accurate diagnosis, risk stratification, and vigilant surveillance. Management is multidisciplinary. In patients with focal bone pain, a palpable tender mass, and imaging suggesting osteolytic destruction, the evaluation should distinguish SBP from MM through biopsy with the confirmation of plasma cell clonality, bone marrow assessment, assessment for myeloma-defining events (CRAB/SLiM criteria), and whole-body imaging (FDG-PET/CT or whole-body MRI). After definitive local therapy—typically radiotherapy—close follow-up with serial serum/urine protein electrophoresis with immunofixation, serum free light chains, and symptom-directed imaging is essential to detect persistent disease and early evolution to myeloma. Risk of progression is higher with residual or rising M-protein, an abnormal free light-chain ratio, minimal marrow involvement by sensitive flow cytometry, larger tumor size, and multifocal lesions on advanced imaging.

## Data Availability

The original contributions presented in the study are included in the article/Supplementary material, further inquiries can be directed to the corresponding authors.
